# Effect of prior antiplatelet therapy on recurrence in patients with acute cerebral ischaemia: data from the triple antiplatelets for reducing dependency after ischaemic stroke (TARDIS) randomised trial

**DOI:** 10.1007/s10072-025-08625-6

**Published:** 2026-01-24

**Authors:** Jason P. Appleton, Lisa J. Woodhouse, Maia Beridze, Hanne Christensen, Rob A. Dineen, Timothy J. England, Anna Ranta, Thompson G. Robinson, Nikola Sprigg, Philip M. Bath

**Affiliations:** 1 Stroke Trials Unit, Mental Health & Clinical Neurosciences, University of Nottingham, South Block D Floor, Queen’s Medical Centre, Nottingham, NG7 2UH UK; 2 Stroke, Nottingham University Hospitals NHS Trust, Nottingham, UK; 3 The First University Clinic of Tblisi, State Medical University, Tbilisi, Georgia; 4 Department of Neurology, University of Copenhagen, Bispebjerg and Frederiksberg Hospital, Copenhagen, Denmark; 5 NIHR Nottingham Biomedical Research Centre, Nottingham, UK; 6 Radiological Sciences, Mental Health & Clinical Neuroscience, School of Medicine, University of Nottingham, Nottingham, UK; 7 Department of Medicine, University of Otago, Wellington, New Zealand; 8 Department of Cardiovascular Sciences and NIHR Biomedical Research Centre, University of Leicester, Leicester, UK

**Keywords:** Acute ischaemic stroke, Transient ischaemic attack, Frailty, Prior antiplatelet, Intensive, Recurrence, Randomised controlled trial

## Abstract

**Background:**

Data regarding management of patients already on an antiplatelet when presenting with an ischaemic stroke or transient ischaemic attack (TIA) are limited. This secondary analysis of the triple antiplatelets for reducing dependency in ischaemic stroke (TARDIS) trial explored clinical outcomes across prior antiplatelet groups.

**Methods:**

TARDIS was an international prospective-randomised open-label blinded-endpoint trial assessing 30 days of triple vs. guideline antiplatelet therapy in patients with acute ischaemic stroke or TIA. The number of pre-stroke/TIA antiplatelet agents was collected pre-randomisation. The primary outcome was the combined incidence of, and dependency from, any recurrent stroke (using the modified Rankin Scale) or TIA within 90 days analysed using ordinal logistic regression with adjustment for prognostic factors. Baseline imaging features of brain frailty were adjudicated centrally by neuroimaging experts.

**Results:**

1080/3096 (34.9%) participants were on an antiplatelet agent prior to their stroke/TIA and were older, more likely to be male, had more co-morbidities and dependency, and more baseline imaging features of brain frailty than those not on prior antiplatelets. They had a higher incidence of, and dependency from, recurrent stroke or TIA at day 90: 86 (8%) vs. 112 (5.6%), adjusted common OR 1.40, 95%CI 1.02–1.92, *p* = 0.036; the result was neutralised when adjusted for brain frailty. Randomisation to triple vs. guideline therapy did not influence this effect.

**Conclusions:**

Participants taking prior antiplatelets were frailer and had a higher incidence and severity of recurrent stroke or TIA at 90 days compared with those not on prior antiplatelets prior to adjustment for brain frailty.

**Trial registration:**

ISRCTN47823388.

**Supplementary Information:**

The online version contains supplementary material available at 10.1007/s10072-025-08625-6.

## Introduction

About a third of patients presenting with minor ischaemic stroke or transient ischaemic attack (TIA) are on an antiplatelet at the time of their index event [1]. Data regarding patients already on an antiplatelet when presenting with an ischaemic stroke or TIA are predominantly limited to subgroups from trials. The definition of prior antiplatelet use and proportion of participants on such treatment varies: in the Clopidogrel in High-risk patients with Acute Non-disabling Cerebrovascular Events (CHANCE) trial, 12% of participants had antiplatelet medication within 24 h before hospital admission [2]; 12% of participants in CHANCE-2 were on antiplatelet therapy within 1 month prior to symptom onset [3]; 15% of participants in the acute stroke or transient ischaemic attack treated with ticagrelor and aspirin for prevention of stroke and death (THALES) trial used an antiplatelet agent before the index event [4]; whilst in the Platelet-Oriented Inhibition in New TIA and minor ischemic stroke (POINT) trial 59% of participants had prior antiplatelet treatment, defined as participant reported use of antiplatelet therapy at the time of qualifying index event prior to randomisation [5].

In the triple antiplatelets for reducing dependency in ischaemic stroke (TARDIS) trial [6], 35% of participants were on an antiplatelet agent pre-index event, akin to that seen in registry data of patients presenting with minor stroke and TIA [1]. Given the paucity of data relating to management of patients presenting with cerebral ischaemia whilst on an antiplatelet, we sought to explore whether baseline data and clinical outcomes differed between prior antiplatelet groups, and assess the treatment effect of triple vs. guideline antiplatelet therapy within this pre-specified subgroup, by performing a secondary analysis of the TARDIS trial.

We hypothesised that:

1. participants on a prior antiplatelet would have more co-morbidities, vascular risk factors, and increased brain imaging markers of ‘brain frailty’.
2. treatment with triple vs. guideline antiplatelet therapy might have a differential effect on recurrent ischaemic events based on prior antiplatelet usage.

## Methods

### Study design

TARDIS was an international, prospective, randomised, open-label, blinded-endpoint (PROBE) trial of triple vs. guideline antiplatelet therapy in patients with non-cardioembolic ischaemic stroke or TIA within 48 h of symptom onset [7–9]. Full inclusion and exclusion criteria are available (protocol available in supplementary material) [6]. In brief, eligible patients were: age ≥ 50 years; non-cardioembolic ischaemic stroke with limb weakness, isolated dysphasia or isolated neuroimaging-positive hemianopia, or a non-cardioembolic TIA with ≥ 10 min of limb weakness or isolated dysphasia; and were randomised within 48 h of symptom onset. Exclusion criteria included: intracranial haemorrhage; non-ischaemic cause for symptoms; pre-event dependency (modified Rankin Scale, mRS > 2); isolated sensory symptoms, facial weakness, vertigo/dizziness; contraindication to, or definite need for, aspirin, clopidogrel, or dipyridamole; and need for anticoagulation. Written informed consent was provided by patients, or from a relative, carer or friend if the patient lacked capacity (example consent form available in supplementary material). The trial was approved by the East Midlands UK research ethics committee (08/H1102/112), registered (ISRCTN47823388), adopted by the National Institute of Health Research (NIHR) Clinical Research Network, and sponsored by the University of Nottingham, UK. National competent authorities gave approvals for the study in each participating country. The trial was overseen by a trial steering committee and an international advisory committee, comprising each national coordinator.

Pre-randomising event antiplatelet therapy was defined as routine administration of one or more of aspirin, clopidogrel, dipyridamole or another antiplatelet taken prior to the index stroke or TIA.

### Patient and public involvement

TARDIS was discussed with, and was supported by, the UK Stroke Research Network Prevention Clinical Studies Group and the Nottingham Stroke Users Research Committee. Two members were patient and public representatives on the trial steering committee during the trial (2008–2016). Public and patient representatives were involved in the design, conduct, reporting, and dissemination of the TARDIS trial.

### Study interventions

Participants were randomised 1:1 to either triple antiplatelet therapy (aspirin 300 mg loading followed by 50–150 mg daily, clopidogrel 300 mg loading followed by 75 mg daily, and dipyridamole modified release 200 mg twice daily) or guideline antiplatelet therapy (clopidogrel alone or aspirin and dipyridamole combined) for 30 days. Guideline antiplatelet therapy was at the discretion of individual sites. After this, participants reverted to local guideline antiplatelet therapy at the discretion of the treating investigator.

### Clinical data

Key characteristics were collected at baseline prior to randomisation including co-morbidities, age, sex, ethnicity, and the number and name(s) of any antiplatelet drugs taken prior to the onset of the index event. Stroke severity was measured using the National Institutes of Health Stroke Scale (NIHSS, ranging from 0 to 42 with higher scores indicating more severe neurological deficit [10]) and risk of recurrence after index TIA assessed using the ABCD2 scale (ranging from 0 to 7 with higher scores indicating higher risk [11]). Clinical syndrome was recorded using the Oxfordshire Community Stroke Project classification [12].

The primary outcome was the incidence of, and dependency from, recurrent stroke and TIA during the 90 day follow-up period assessed as a 6-level ordinal scale: fatal stroke; non-fatal stroke (mRS 4 or 5); moderate stroke (mRS 2 or 3); mild stroke (mRS 0 or 1); TIA; and no stroke or TIA [6]. Day 90 secondary outcomes included: disability measured using Barthel index; cognition using modified telephone mini-mental state examination (t-MMSE), telephone interview for cognition scale-modified (TICS-M), and verbal fluency using animal naming; mood using Zung depression score (ZDS); and quality of life using health status utility value (HSUV) derived from the European quality of life-5 dimensions-3 level (EQ-5D-3L), and visual analogue scale (EQ-VAS).

Safety outcomes included bleeding events (defined according to the International Society on Thrombosis and Haemostasis [13], based on severity, site of bleeding, haemoglobin fall and need for transfusion), death and serious adverse events (available in supplement of main trial publication) [6]. Composite outcomes included: stroke or major/fatal bleeding; and death, stroke, myocardial infarction, or major bleeding.

At days 7 and 35 participants were seen to record treatment compliance and any clinical outcome or bleeding events. Final follow-up was performed centrally by telephone at 90 days by blinded trained assessors. A paper version was sent by post if participants couldn’t be reached by telephone. Thus, recurrent cerebrovascular event data were captured via the following methods: at days 7 and 35 by site investigators; in any serious adverse events; at day 90 final follow-up; and in a questionnaire sent to general practitioners after day 90. Primary outcome, haemorrhage and serious adverse event data were adjudicated by experts blinded to treatment assignment.

### Neuroimaging data

Neuroimaging was required for all ischaemic stroke patients prior to inclusion in TARDIS, and although this wasn’t a necessity for TIA patients, the majority underwent imaging too. Computerised topography (CT) or magnetic resonance imaging (MRI) scans were performed according to local clinical practice and, along with any additional clinical scans, were collected and adjudicated centrally by a trained panel of expert neuroradiologists blinded to symptoms and randomised treatment using validated forms [14, 15]. CT was the commonest imaging modality used and whilst MRI was subsequently performed in 776/3096 (25.1%) participants, the first scan was CT-based for the majority and adjudicated as the baseline scan.

Background imaging changes including cerebral atrophy, periventricular white matter lucencies (leukoaraiosis) and old vascular lesion(s) were recorded from available imaging (either CT or MRI) as done previously [14, 15]. Cerebral atrophy was scored in cortical and central areas as 0 = absent, 1 = moderate, 2 = severe, giving a maximum score of 4; periventricular white matter lucencies were scored in anterior and posterior regions as 0 = absent, 1 = restricted to region adjoining ventricles, 2 = lucency covering lateral ventricle to cortex, giving a maximum score of 4; old vascular lesion recorded by location (cortical, striatocapsular, borderzone, lacunar, brainstem/cerebellar). These background changes were amalgamated as a brain frailty score [15]: 1 point for scores of 1 or 2 cortical and/or central cerebral atrophy; 1 point for scores of 1 or 2 anterior and/or posterior periventricular white matter lucencies; and 1 point for any old vascular lesion, giving a maximum brain frailty score of 3.

### Sample size calculation

For the overall TARDIS trial, the estimated sample size was 4100 participants to detect a shift in the distribution of the primary outcome using an ordinal analysis with a common odds ratio 0.68, 5% two-sided type I error, 90% power, 2% dropout frequency, 5% treatment crossover, and adjustment for baseline covariates [6, 8]. The TARDIS trial was stopped early due to futility after recruiting 3096 participants (76% of the planned 4100) [6].

### Statistical Analysis

In line with the TARDIS trial statistical analysis plan and statistical analyses performed in the primary publication, data were analysed by intention-to-treat [6, 8]. Data are number (%), median [interquartile range, IQR], or mean (standard deviation, SD). Baseline characteristics between prior antiplatelet or no antiplatelet groups were assessed using X^2^ for categorical variables and one-way analysis of variance (ANOVA) for continuous variables.

Associations between prior antiplatelet use and outcomes were assessed using binary logistic regression (producing adjusted odds ratio [aOR]), ordinal logistic regression (adjusted commons odds ratio [acOR]) or multiple linear regression (adjusted mean difference [aMD]), with adjustment for baseline covariates including: index event (TIA vs stroke), country, guideline randomisation choice, age, sex, pre-morbid function, systolic blood pressure, stroke syndrome (cortical vs. lacunar [12]), use of gastroprotection, use of low dose heparin, time to randomisation, NIHSS, ABCD2 score, number of TIAs in the last week and treatment with alteplase. A post-hoc sensitivity analysis including brain frailty was performed due to baseline imbalance between prior antiplatelet groups. Analyses involving the whole population were also adjusted for treatment allocation. Interaction p-values were obtained by adding an interaction term to statistical models. 95% confidence intervals (CI) are given, with significance defined as *p* ≤ 0.05. Analyses were performed using SPSS version 29 (Chicago, IL USA).

## Results

### Baseline characteristics

Between April 2009 and March 2016, TARDIS recruited 3096 participants at 106 sites in four countries (Denmark, Georgia, New Zealand, UK). A full list of participating sites and a participant flow diagram are available in the main publication. [6] i.e Of these, 1080 (34.9%) participants were on an antiplatelet agent prior to their index event and they were older, more likely to be male, recruited in the UK, and have a pre-morbid mRS > 1, hypertension, diabetes, previous stroke, ischaemic heart disease, peripheral arterial disease or hyperlipidaemia, and be taking gastroprotection; conversely, they were less likely to be current smokers than those not on a prior antiplatelet agent (Table 1). Most participants who were taking a single prior antiplatelet were on aspirin (83.2%), with the remainder on clopidogrel (16.5%) or dipyridamole (0.3%). Of those on dual antiplatelets (99/3096, 3%) prior to their index event, most (86.9%) were on aspirin and dipyridamole, with a smaller proportion on aspirin and clopidogrel (11%). There was no association between prior antiplatelet groups and the trial index event type of ischaemic stroke versus TIA. Similarly, NIHSS, time to randomisation and the proportion who underwent thrombolysis did not differ between groups. The ABCD2 score was higher in those on a prior antiplatelet, they had lower mean blood pressure (BP), were more likely to have a large vessel event and total anterior circulation syndrome than those not on a prior antiplatelet. Those on a prior antiplatelet had more cerebral atrophy, leukoaraiosis, old vascular lesion(s) and higher brain frailty scores on neuroimaging than those not on a prior antiplatelet (Table 1).

**Table 1:** Baseline characteristics of all patients by prior antiplatelet usage

	All	Prior antiplatelet		*p*	Prior antiplatelet			*p*
		Yes	No		0	1	> 1	
Number of patients	3096	1080	2016		2016	981	99	
Age (years) †	69.0 (10.1)	71.8 (9.7)	67.5 (10.0)	** < 0.001**	67.5 (10.0)	71.8 (9.8)	71.4 (9.6)	** < 0.001**
Sex, male (%) †	1945 (62.8)	727 (67.3)	1218 (60.4)	** < 0.001**	1218 (60.4)	655 (66.8)	72 (72.7)	** < 0.001**
Premorbid mRS > 1 (%) †	122 (3.9)	79 (7.3)	43 (2.1)	** < 0.001**	43 (2.1)	72 (7.3)	7 (7.1)	** < 0.001**
Country, UK (%) †	2955 (95.4)	1044 (96.7)	1911 (94.8)	**0.017**	1911 (94.8)	946 (96.4)	98 (99.0)	**0.030**
Time to randomisation (hours), [IQR] †	29.3 [21.8, 39.6]	29.3 [21.6, 39.1]	29.3 [21.8, 39.7]	0.82	29.3 [21.8, 39.7]	29.3 [21.7, 39.5]	27.7 [19.5, 34.6]	0.22
*Randomisation choices*								
Guideline choice, AD (%) †	1116 (36.0)	411 (36.8)	705 (35.0)	0.09	705 (35.0)	344 (35.1)	67 (67.7)	** < 0.001**
Use of low dose heparin, yes †	6 (0.2)	4 (0.4)	2 (0.1)	0.10	2 (0.1)	4 (0.4)	0	0.18
Gastroprotection, yes (%) †	803 (25.9)	384 (35.6)	419 (20.8)	** < 0.001**	419 (20.8)	345 (35.2)	39 (39.4)	** < 0.001**
***Medical history (%)***								
Previous antiplatelets ‡	1080 (34.9)							
Aspirin	914 (29.5)	914 (84.6)	0	** < 0.001**	0	816 (83.2)	98 (99.0)	** < 0.001**
Clopidogrel	174 (5.6)	174 (16.1)	0	** < 0.001**	0	162 (16.5)	12 (12.1)	** < 0.001**
Dipyridamole	90 (2.9)	90 (8.3)	0	** < 0.001**	0	3 (0.3)	87 (87.9)	** < 0.001**
Aspirin/dipyridamole	86 (2.8)	86 (8.0)	0	** < 0.001**	0	0	86 (86.9)	** < 0.001**
Aspirin/clopidogrel	11 (0.4)	11 (0.4)	0	** < 0.001**	0	0	11 (11.1)	** < 0.001**
Hypertension	1824 (58.9)	872 (80.7)	952 (47.2)	** < 0.001**	952 (47.2)	799 (81.4)	73 (73.7)	** < 0.001**
Diabetes mellitus	590 (19.1)	312 (28.9)	278 (13.8)	** < 0.001**	278 (13.8)	288 (29.4)	24 (24.2)	** < 0.001**
Atrial fibrillation	1 (0.0)	1 (0.1)	0	0.17	0	1 (0.1)	0	0.34
Stroke	348 (11.2)	277 (25.6)	71 (3.5)	** < 0.001**	71 (3.5)	222 (22.6)	55 (55.6)	** < 0.001**
Ischaemic heart disease	403 (13.0)	346 (32.0)	57 (2.8)	** < 0.001**	57 (2.8)	321 (32.7)	25 (25.3)	** < 0.001**
Peripheral arterial disease	70 (2.3)	51 (4.7)	19 (0.9)	** < 0.001**	19 (0.9)	50 (5.1)	1 (1.0)	** < 0.001**
Hyperlipidaemia	1317 (42.5)	758 (70.2)	559 (27.7)	** < 0.001**	559 (27.7)	685 (69.8)	73 (73.7)	** < 0.001**
Smoking, current	784 (25.3)	220 (20.4)	564 (28.0)	** < 0.001**	564 (28.0)	202 (20.6)	18 (18.2)	** < 0.001**
*Randomising event* †								
Ischaemic stroke (%)	2143 (69.2)	734 (68.0)	1409 (69.9)	0.27	1409 (69.9)	678 (69.1)	56 (56.6)	**0.020**
TIA (%)	953 (30.8)	346 (32.0)	607 (30.1)	0.27	607 (30.1)	303 (30.9)	43 (43.4)	**0.020**
Crescendo TIA † (%)	184 (5.9)	57 (5.3)	127 (6.3)	0.25	127 (6.3)	48 (4.9)	9 (9.1)	0.13
*Clinical*								
NIHSS (/42) †	2.8 (3.6)	2.9 (3.8)	2.8 (3.5)	0.49	2.8 (3.5)	2.9 (3.9)	2.5 (3.2)	0.39
ABCD (/7) †	5.4 (0.9)	5.5 (0.9)	5.3 (0.8)	** < 0.001**	5.3 (0.8)	5.5 (0.9)	5.4 (0.9)	** < 0.001**
TOAST classification* (%)								
Cardioembolic	145 (4.7)	57 (5.3)	88 (4.4)	0.18	88 (4.4)	50 (5.1)	7 (7.1)	0.36
Large vessel	505 (16.3)	207 (19.2)	298 (14.8)	**0.002**	298 (14.8)	185 (18.9)	22 (22.2)	**0.010**
Small Vessel	1242 (40.1)	388 (35.9)	854 (42.4)	**0.001**	854 (42.4)	357 (36.4)	31 (31.3)	**0.006**
Other	1188 (38.4)	423 (39.2)	765 (37.9)	0.27	765 (37.9)	386 (39.3)	37 (37.4)	0.57
Syndrome, TACS (%) †	181 (5.8)	76 (7.0)	105 (5.2)	**0.039**	105 (5.2)	71 (7.2)	5 (5.1)	0.08
Blood pressure (mmHg)								
Systolic (mmHg) †	143.5 (18.2)	141.5 (17.9)	144.6 (18.2)	** < 0.001**	144.6 (18.2)	141.6 (17.8)	140.3 (18.7)	** < 0.001**
Diastolic (mmHg)	79.5 (11.4)	76.9 (10.7)	80.9 (11.5)	** < 0.001**	80.9 (11.5)	76.9 (10.7)	77.1 (11.1)	** < 0.001**
Thrombolysis (%) †	341 (11.0)	112 (10.4)	229 (11.4)	0.40	229 (11.4)	103 (10.5)	9 (9.1)	0.64
*Baseline imaging*								
Cerebral atrophy score [/4]	1 [1, 2]	2 [1, 2]	1 [1, 2]	** < 0.001**	1 [1, 2]	2 [1, 2]	2 [1, 3]	** < 0.001**
Leukoaraiosis score [/4]	0 [0, 1]	0 [0, 2]	0 [0, 1]	** < 0.001**	0 [0, 1]	0 [0, 2]	0 [0, 2]	** < 0.001**
Old vascular lesion (%)	1707 (58.2)	669 (66.1)	1038 (54.1)	** < 0.001**	1038 (54.1)	612 (66.3)	57 (64)	** < 0.001**
Brain frailty score [/3]	1 [2]	2 [1, 3]	2 [1, 2]	** < 0.001**	2 [1, 2]	2 [1, 3]	2 [1, 3]	** < 0.001**

*total may exceed 100% due to mixed causality. *AD* aspirin + dipyridamole, *BP* blood pressure, *IHD* ischaemic heart disease, *IQR* interquartile range, *mRS* modified rankin scale, *NIHSS* national institutes of health stroke scale, *PAD* peripheral arterial disease, *TIA* transient ischaemic attack, *TOAST* trial of org 10172 in acute stroke treatment. Comparison across prior antiplatelet groups by Chi-square test, Kruskal–Wallis test, or one-way analysis of variance (ANOVA)

† Minimisation variables used at randomisation

‡ Variable defining analysis groups

### Relationship between prior antiplatelet use and outcome

**Fig. 1:**
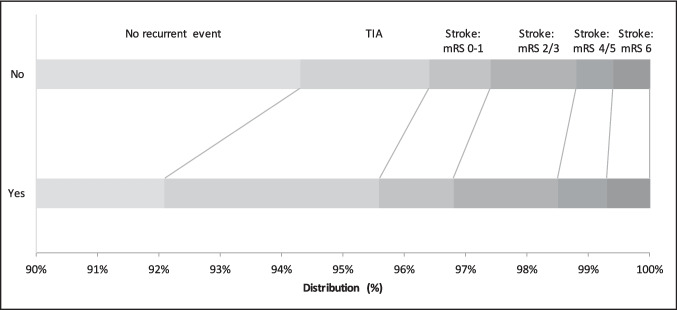
Recurrent cerebrovascular event (using mRS) at day 90 by prior antiplatelet use. mRS: modified Rankin Scale; TIA: transient ischaemic attack

Those on a prior antiplatelet had a higher incidence of, and dependency from, recurrent stroke or TIA at day 90 compared with those not on a prior antiplatelet: 86/1075 (8.0%) vs. 112/1995 (5.6%), acOR 1.40, 95% CI 1.02–1.92, *p* = 0.036 (Table 2, Fig. 1). When brain frailty was added to the ordinal logistic regression model the effect seen above was neutralised: acOR 1.27, 95% CI 0.90–1.79, *p* = 0.17. Participants on a prior antiplatelet had worse mood scores and quality of life scores on the EQ-VAS at day 90 compared with those not on a prior antiplatelet, and there was a tendency towards increased incidence of the composite outcome of death, stroke, myocardial infarction, or major bleeding as compared with those not on a prior antiplatelet (Table 2).

**Table 2:** Primary and secondary outcomes at day 90 by prior antiplatelet status

	Prior antiplatelets			OR/cOR/MD (95% CI)	*p*
	Yes	No			
Ordinal stroke or TIA (%), *n* = 3070	86 (8.0)	112 (5.6)		1.40 (1.02, 1.92)	**0.036**
No stroke or TIA	989 (92.0)	1883 (94.4)		-	-
TIA	38 (3.5)	42 (2.1)		-	-
mRS 0–1	13 (1.2)		20 (1.0)	-	-
mRS 2–3	18 (1.7)		27 (1.4)	-	-
mRS 4–5	9 (0.8)		11 (0.6)	-	-
mRS 6 (death)	8 (0.7)		12 (0.6)	-	-
Stroke (%)	48 (4.5)		70 (3.5)	1.31 (0.88, 1.97)	0.19
Death (%), *n* = 3096	26 (2.4)		28 (1.4)	1.54 (0.85, 2.80)	0.15
Barthel index, *n* = 2980	91.5 (20.5)		93.5 (18.5)	−0.70 (−2.08, 0.67)	0.32
ZDS, *n* = 2506	47.6 (17.9)		45.7 (16.9)	1.61 (0.18, 3.03)	**0.028**
EQ-5D-3L-HSUV, *n* = 2991	0.72 (0.31)		0.76 (0.30)	−0.02 (−0.04, 0.01)	0.14
EQ-VAS, n = 2859	69.5 (22.9)		73.9 (21.3)	−2.83 (−4.54, −1.13)	**0.001**
t-MMSE, *n* = 2360	17.9 (4.7)		18.6 (4.1)	−0.23 (−0.60, 0.14)	0.22
TICS-M, *n* = 2389	20.2 (6.5)		21.5 (6.2)	−0.43 (−0.96, 0.10)	0.11
Verbal fluency, *n* = 2412	16.2 (7.4)		17.4 (7.6)	−0.28 (−0.90, 0.34)	0.37
Intracranial bleeding	10 (0.9)		11 (0.6)	1.68 (0.67, 4.24)	0.27
Stroke or major/fatal bleeding	63 (5.9)		93 (4.7)	1.26 (0.88, 1.79)	0.21
Death, stroke, myocardial infarction, or major bleeding	86 (8)		114 (5.7)	1.35 (0.99, 1.85)	0.060

Data are *n* (%), mean (standard deviation), mean difference (MD), odds ratio (OR) or common odds ratio (cOR) with 95% confidence intervals (CI). Comparison using binary or ordinal logistic regression or multiple linear regression. *EQ-5D-3L-HSUV* European quality of life 5 dimensions 3 level health utility status value, *EQ-VAS* European quality of life visual analogue scale, *mRS* modified rankin scale, *t-MMSE* telephone mini-mental state examination, *TIA* transient ischaemic attack, *TICS-M* telephone interview for cognition scale-modified, *ZDS* Zung depression scale

When compared with those not on a prior antiplatelet, those on one prior antiplatelet had a higher incidence of, and dependency from, recurrent stroke or TIA at 90 days (77/976 (7.9%) vs. 112/1995 (5.6%), acOR 1.40, 95% CI 1.01–1.93, *p* = 0.041), worse quality of life scores on EQ-VAS, and a higher incidence of the composite outcome death, stroke, myocardial infarction, or major bleeding (Supplementary Table 1). There were no differences in other secondary outcomes between prior antiplatelet groups (Supplementary Table 1).

### Effects of triple vs. guideline antiplatelet therapy

**Fig. 2:**
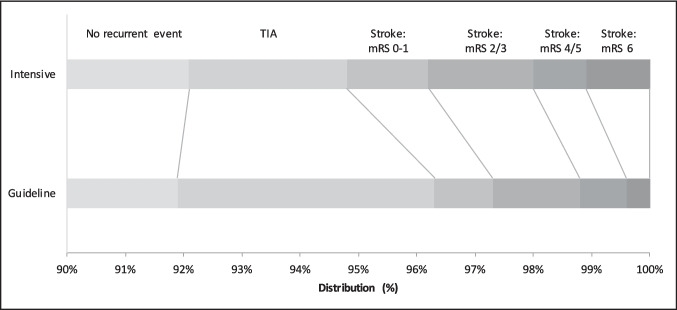
Recurrent cerebrovascular event (using mRS) at day 90 in those with prior antiplatelet use by intensive vs. guideline antiplatelet therapy. mRS: modified Rankin Scale; TIA: transient ischaemic attack

**Fig. 3:**
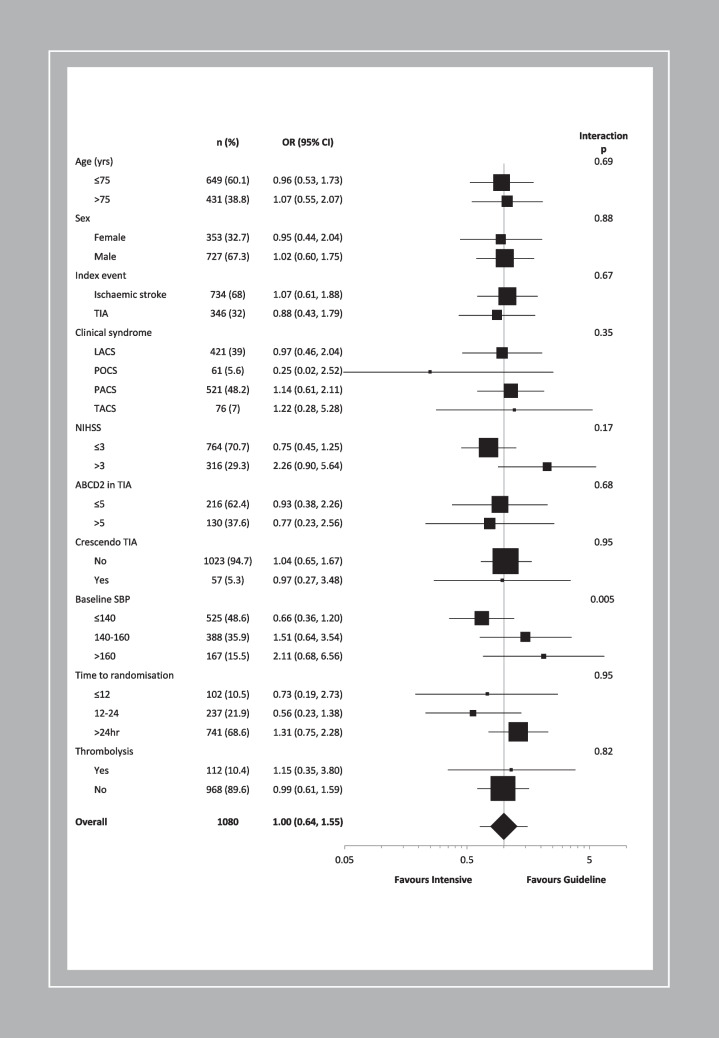
Forest plot of recurrent cerebrovascular event (using mRS) at day 90 by intensive vs. guideline antiplatelet therapy in those on prior antiplatelets. Unadjusted ordinal logistic regression. LACS: lacunar syndrome; mRS: modified Rankin scale; NIHSS: National Institutes of Health Stroke Scale; OR: odds ratio; PACS: partial anterior circulation syndrome; POCS: posterior circulation syndrome; SBP: systolic blood pressure; TACS: total anterior circulation syndrome; TIA: transient ischaemic attack

In those on a prior antiplatelet, triple vs. guideline antiplatelet therapy did not influence the incidence of, and dependency from, recurrent stroke or TIA at 90 days: 44/555 (7.9%) vs. 42/520 (8.1%), acOR 1.08, 95% CI 0.69–1.69, 0.75 (Table 3, Fig. 2). In pre-defined subgroups, there was a significant interaction between baseline systolic BP and triple vs. guideline antiplatelet therapy on the primary outcome in those on a prior antiplatelet (*p* = 0.005, Fig. 3). Those with baseline systolic BP < 140 mmHg had a point estimate in favour of triple antiplatelet therapy, whilst those with systolic BP 140–160 mmHg and > 160 mmHg had point estimates in favour of guideline antiplatelet therapy. There were no other significant interactions seen.

**Table 3:** Primary and secondary outcomes at day 90 by randomised treatment and prior antiplatelet use

	No prior antiplatelets					One or more prior antiplatelets			
	ACD	Guideline		OR/cOR/MD (95% CI)	*p*	ACD	Guideline	OR/cOR/MD (95% CI)	*p*
Ordinal stroke or TIA (%)	49 (5.0)	63 (6.2)		0.81 (0.55, 1.19)	0.28	44 (7.9)	42 (8.1)	1.08 (0.69, 1.69)	0.75
No stroke or TIA	936 (95.0)	947 (93.8)		-	-	511 (92.1)	478 (91.9)	-	-
TIA	17 (1.7)	25 (2.5)		-	-	15 (2.7)	23 (4.4)	-	-
mRS 0–1	7 (0.7)		13 (1.3)	-	-	8 (1.4)	5 (1.0)	-	-
mRS 2–3	12 (1.2)		15 (1.5)	-	-	10 (1.8)	8 (1.5)	-	-
mRS 4–5	6 (0.6)		5 (0.5)	-	-	5 (0.9)	4 (0.8)	-	-
mRS 6 (death)	7 (0.7)		5 (0.5)	-	-	6 (1.1)	2 (0.4)	-	-
Stroke (%)	32 (3.2)		38 (3.8)	0.85 (0.53, 1.38)	0.52	29 (5.2)	19 (3.7)	1.54 (0.84, 2.80)	0.16
Death (%)	15 (1.5)		13 (1.3)	1.08 (0.50, 2.35)	0.85	11 (2.0)	15 (2.9)	0.70 (0.31, 1.62)	0.41
Barthel Index	92.9 (19.6)		94.2 (17.4)	−0.94 (−2.40, 0.53)	0.21	91.6 (20.1)	91.4 (20.8)	−0.27 (−2.53, 1.98)	0.81
ZDS	45.4 (16.8)		45.9 (17.1)	−0.37 (−1.93, 1.20)	0.65	47.4 (17.4)	47.9 (18.3)	−0.20 (−2.48, 2.08)	0.86
EQ-5D-3L-HSUV	0.76 (0.31)		0.76 (0.29)	0.01 (−0.02, 0.03)	0.68	0.73 (0.32)	0.71 (0.31)	0.02 (−0.03, 0.05)	0.48
EQ-VAS	74.3 (21.0)		73.6 (21.6)	0.81 (−1.03, 2.65)	0.39	69.9 (22.3)	69.0 (23.5)	0.75 (−2.00, 3.51)	0.59
t-MMSE	18.5 (4.2)		18.7 (3.9)	−0.22 (−0.61, 0.16)	0.26	18.1 (4.2)	17.7 (5.3)	0.35 (−0.27, 0.98)	0.27
TICS-M	21.4 (6.4)		21.7 (6.0)	−0.26 (−0.84, 0.32)	0.37	20.4 (6.0)	19.9 (7.1)	0.59 (−0.25, 1.44)	0.17
Verbal fluency	17.3 (7.7)		17.6 (7.6)	−0.26 (−0.95, 0.43)	0.46	16.5 (7.5)	15.8 (7.4)	0.58 (−0.36, 1.52)	0.22
Intracranial bleeding	7 (0.7)		4 (0.4)	1.84 (0.52, 6.50)	0.34	9 (1.6)	1 (0.2)	11.52 (1.31, 101.69)	**0.028**
Stroke or major/fatal bleeding	47 (4.8)		46 (4.6)	1.00 (0.61, 1.64)	1.00	40 (7.2)	23 (4.4)	1.80 (0.92, 3.53)	0.09
Death, stroke, myocardial infarction, or major bleeding	56 (5.7)		58 (5.7)	1.04 (0.66, 1.66)	0.86	46 (8.3)	40 (7.7)	1.03 (0.60, 1.76)	0.93

Data are *n* (%), mean (standard deviation), mean difference (MD), odds ratio (OR) or common odds ratio (cOR) with 95% confidence intervals (CI). Comparison using binary or ordinal logistic regression or multiple linear regression. *ACD* aspirin, clopidogrel, dipyridamole, *EQ-5D-3L-HSUV* European quality of life 5 dimensions 3 level health utility status value, *EQ-VAS* European quality of life visual analogue scale, *mRS* modified rankin scale, *t-MMSE* telephone mini-mental state examination, *TIA* transient ischaemic attack, *TICS-M* telephone interview for cognition scale-modified, *ZDS* Zung depression scale

Triple vs. guideline antiplatelet therapy was associated with more intracranial bleeding in those on a prior antiplatelet, although the number of bleeds was small. Randomised treatment did not influence other secondary outcomes regardless of prior antiplatelet status (Table 3, Supplementary Table 2).

## Discussion

In this secondary analysis of the TARDIS trial, 35% of participants were on an antiplatelet prior to their presenting event; they had more co-morbidities and imaging markers of brain frailty than those not on a prior antiplatelet, had a higher incidence of, and dependency from, recurrent stroke or TIA at 90 days (primary outcome), and worse mood and quality of life scores despite adjustment for baseline prognostic markers. Additional adjustment for brain frailty neutralised the effect seen on the primary outcome. Prior antiplatelet use represents a marker of vascular disease and vascular risk, rather than being a direct cause of recurrent events. Randomisation to triple vs. guideline antiplatelet therapy was associated with more intracranial bleeding in those on a prior antiplatelet, but did not influence other outcomes including composite outcomes combining stroke and bleeding events.

The definition of prior antiplatelet use has varied in recent trials assessing dual antiplatelet therapy (DAPT) in minor ischaemic stroke and TIA, which may, in part, explain why there have been conflicting results reported on the influence of prior antiplatelet use on the efficacy of randomised treatment. In the POINT trial, 59% of participants had antiplatelet pre-treatment and when compared to those with no antiplatelet pre-treatment, they were older, had more co-morbidities, and were less likely to be current smokers – similar findings to the present analysis, although they didn’t find an association with recurrent ischaemic stroke at 90 days and did not consider adjustment for baseline neuroimaging features [5]. There was no difference in the treatment effect of DAPT on reducing ischaemic stroke at 90 days. In contrast, 15% of participants in THALES were on an antiplatelet agent before the index event and they found that DAPT was associated with stroke reduction only in those not on a prior antiplatelet [4]. In CHANCE, 12% of participants had antiplatelet medication within 24 h before hospital admission and found no interaction between prior antiplatelet use and DAPT on outcome [2]. Prior antiplatelet use represents a marker of vascular disease, increased vascular risk and co-morbidity as patients are initiated on an antiplatelet due to their vascular history, which would explain the increased risk of recurrent events seen in this subgroup analysis. Importantly, antiplatelets may not work in all patients e.g. due to poor adherence, lack of absorption, genetic polymorphisms involved in metabolism [16].

Brain frailty was common in TARDIS overall, was more prevalent in those on a prior antiplatelet compared with those not, and neutralised the effect of prior antiplatelet usage on the primary outcome when added to a statistical model already adjusted for baseline prognostic factors, including mRS. This finding builds on prior work demonstrating the prognostic value of baseline imaging markers of brain frailty in acute stroke, including the independent associations with functional outcome, death, cognition, quality of life, and mood disturbance after stroke [14, 15, 17, 18]. As such, stroke trialists and statisticians may wish to include baseline brain frailty as a minimisation variable or at least consider adjusting for this in statistical models in acute stroke and TIA trials. Given the challenges and inaccuracies with using baseline mRS as a marker of pre-stroke function, brain frailty could be used as a surrogate. Clinicians may wish to measure brain frailty on baseline imaging and consider its impact on prognosis after TIA and stroke. Escalation to triple antiplatelets in a frailer population already on an antiplatelet did not reduce recurrent ischaemic events, but did increase intracranial bleeding, and so this approach is probably best avoided pending further data. Whether brain frailty and frailty more generally should influence prescribing in acute cerebral ischaemia warrants further research.

There was a significant interaction between baseline systolic BP and randomised antiplatelet therapy on the primary outcome in those on a prior antiplatelet; those with a systolic BP < 140 mmHg had a point estimate in favour of triple antiplatelet therapy, whilst higher BP groups had point estimates in favour of guideline antiplatelet therapy. This is in contrast to secondary analyses of CHANCE [19] and the Antiplatelet Therapy in Acute Mild to Moderate Ischemic Stroke (ATAMIS) trials [20], that found that in patients with baseline systolic BP > 140 mmHg treatment with DAPT reduced the risk of stroke recurrence and early neurological deterioration, respectively; an effect not seen in those with systolic BP < 140 mmHg. Therefore, our finding may represent chance.

This secondary analysis of the TARDIS trial has limitations. First, prior antiplatelet information was collected by sites and not corroborated centrally nor cross-checked with pharmacy or GP records. This may have introduced bias, under-estimated non-adherence or over-estimated exposure, but provides generalisability to clinical practice. Second, the definition of prior antiplatelet use varies in the literature and may explain why our results are similar to some trial subgroup analyses but not others. Third, neuroimaging was not mandated for TIA participants (in line with clinical practice at the time) and so there may be participants labelled as TIA who may have had diffusion-positive events on MRI that may be categorised as stroke on the tissue rather than time approach. Baseline imaging markers of brain frailty were assessed on available imaging (either CT or MRI, but predominantly CT) allowing generalisability to clinical practice. Although CT lacks the sensitivity of MRI for some more subtle features of small vessel disease, pooled scores of small vessel disease and brain frailty may represent differing aspects of brain health and have differential effects on outcome after acute stroke [15, 17]. However, direct CT vs. MRI comparisons are awaited. Fourth, as a subgroup analysis without adjustment for multiplicity of testing, the findings may represent chance. Last, TARDIS was stopped early for futility after enrolling 76% of the planned sample size. As such, the absence of significant associations in some subgroup analyses should be interpreted with caution, as they may simply reflect insufficient power rather than true lack of effect.

In summary, in this secondary analysis of the TARDIS trial, 35% of participants were on an antiplatelet prior to their presenting event and had more co-morbidities and imaging markers of brain frailty than those not on a prior antiplatelet. Despite adjustment for baseline prognostic markers, those participants on a prior antiplatelet had a higher incidence of, and dependency from, recurrent stroke or TIA at 90 days, but following additional adjustment for brain frailty, this effect was no longer seen. Although prior antiplatelet use is an indicator of vascular disease, brain frailty is a strong predictor of outcome after acute stroke and therefore future acute stroke and TIA trials may wish to consider this in their design and statistical analysis plans and it may become a useful prognostic marker for clinicians in future.

## Supplementary Information

Below is the link to the electronic supplementary material.

Supplementary file1 (DOCX 31 KB)

## Data Availability

Supplementary material available online. Data pertaining to the presented analyses are available from the corresponding author upon reasonable request with an appropriate protocol for planned analyses.

## Abbreviations

CI: Confidence interval
MD: Mean difference
mRS: Modified Rankin Scale
OR: Odds ratio
TARDIS: Triple Antiplatelets for Reducing Dependency after Ischaemic Stroke
