# Supplementary material for: Effect of prior antiplatelet therapy on recurrence in patients with acute cerebral ischaemia: data from the triple antiplatelets for reducing dependency after ischaemic stroke (TARDIS) randomised trial
Source: Neurol Sci. 2026 Jan 24;47(2):191. doi: 10.1007/s10072-025-08625-6 (PMC12831681; doi:10.1007/s10072-025-08625-6)
Supplement: Supplementary file 1 — Supplementary file1 (DOCX 31 KB) [file 10072_2025_8625_MOESM1_ESM.docx]

**Effect of prior antiplatelet therapy on recurrence in patients with acute cerebral ischaemia: data from the Triple Antiplatelet for Reducing Dependency after Ischaemic Stroke (TARDIS) randomised trial**

**SUPPLMENTARY MATERIAL**

**Supplementary Table 1: Primary and secondary outcomes at day 90 by number of prior antiplatelet agents**

|  | **No prior antiplatelets** | **One prior antiplatelet** | | | **More than 1 antiplatelet** | | |
| --- | --- | --- | --- | --- | --- | --- | --- |
|  | **n (%) / mean (SD)** | **n (%) / mean (SD)** | **OR/cOR/MD (95% CI)** | **p** | **n (%) / mean (SD)** | **OR/cOR/MD (95% CI)** | **p** |
| Number of Participants | 1995 | 976 | - | - | 99 | - | - |
| Ordinal stroke or TIA (%) | 112 (5.6) | 77 (7.9) | 1.40 (1.01, 1.93) | **0.041** | 9 (9.1) | 1.43 (0.67, 3.05) | 0.35 |
| No stroke or TIA | 1883 (94.4) | 899 (92.1) | - | - | 90 (90.9) | - | - |
| TIA | 42 (2.1) | 30 (3.1) | - | - | 8 (8.1) | - | - |
| mRS 0-1 | 20 (1.0) | 13 (1.3) | - | - | 0 | - | - |
| mRS 2-3 | 27 (1.4) | 17 (1.7) | - | - | 1 (1.0) | - | - |
| mRS 4-5 | 11 (0.6) | 9 (0.9) | - | - | 0 | - | - |
| mRS 6 (death) | 12 (0.6) | 8 (0.8) | - | - | 0 | - | - |
| Stroke (%) | 70 (3.5) | 47 (4.8) | 1.42 (0.95, 2.12) | 0.09 | 1 (1.0) | 0.28 (0.04, 2.05) | 0.21 |
| Death (%) | 28 (1.4) | 25 (2.5) | 1.61 (0.88, 2.94) | 0.12 | 1 (1.0) | 0.75 (0.10, 5.83) | 0.78 |
| Barthel Index | 93.5 (18.5) | 91.4 (20.8) | 0.78 (-0.94, 2.50) | 0.83 | 92.5 (17.5) | -0.09 (-4.42, 4.25) | 1.00 |
| ZDS | 45.7 (16.9) | 47.6 (17.8) | -1.66 (-3.46, 0.14) | 0.08 | 47.8 (18.7) | -1.66 (-6.13, 2.81) | 1.00 |
| EQ-5D-3L-HSUV | 0.76 (0.30) | 0.72 (0.32) | 0.02 (-0.01, 0.05) | 0.27 | 0.74 (0.29) | 0.00 (-0.07, 0.07) | 1.00 |
| EQ-VAS | 73.9 (21.3) | 69.5 (23.0) | 2.92 (0.77, 5.06) | **0.003** | 69.4 (21.3) | 2.57 (-2.85, 8.00) | 0.77 |
| t-MMSE | 18.6 (4.1) | 17.9 (4.8) | 0.21 (-0.25, 0.67) | 0.84 | 17.7 (4.2) | 0.46 (-0.74, 1.65) | 1.00 |
| TICS-M | 21.5 (6.2) | 20.2 (6.6) | 0.43 (-0.24, 1.10) | 0.37 | 20.0 (5.8) | 0.47 (-1.20, 2.14) | 1.00 |
| Verbal fluency | 17.4 (7.6) | 16.3 (7.4) | 0.15 (-0.65, 0.95) | 1.00 | 15.3 (7.7) | 1.49 (-0.49, 3.48) | 0.21 |
| Intracranial bleeding | 11 (0.6) | 10 (1.0) | 1.82 (0.73, 4.58) | 0.20 | 0 | NC |  |
| Stroke or major/fatal bleeding | 93 (4.7) | 60 (6.1) | 1.33 (0.93, 1.90) | 0.12 | 3 (3) | 0.61 (0.19, 1.98) | 0.41 |
| Death, stroke, myocardial infarction, or major bleeding | 114 (5.7) | 83 (8.5) | 1.44 (1.05, 1.98) | **0.024** | 3 (3) | 0.48 (0.15, 1.56) | 0.22 |

Data are n (%), mean (standard deviation), mean difference (MD), odds ratio (OR) or common odds ratio (cOR) with 95% confidence intervals (CI). Comparison using binary or ordinal logistic regression or multiple linear regression with ‘no antiplatelet’ group as reference group. EQ-5D-3L-HSUV: European quality of life 5 dimensions 3 level health utility status value; EQ-VAS: European quality of life visual analogue scale; mRS: modified Rankin Scale; t-MMSE: telephone mini-mental state examination; TIA: transient ischaemic attack; TICS-M: telephone interview for cognition scale-modified; ZDS: Zung Depression Scale.

**Supplementary Table 2: Primary and secondary outcomes at day 90 by randomised treatment and number of prior antiplatelets**

|  | **No prior antiplatelets** | | | | | | | **One prior antiplatelet** | | | | **More than 1 antiplatelet** | | | |
| --- | --- | --- | --- | --- | --- | --- | --- | --- | --- | --- | --- | --- | --- | --- | --- |
|  | **ACD** | | **Guideline** | | **OR/cOR/MD (95% CI)** | | **p** | **ACD** | **Guideline** | **OR/cOR/MD  (95% CI)** | **p** | **ACD** | **Guideline** | **OR/cOR/MD  (95% CI)** | **p** |
| Number of Participants | 999 | | 1017 | | - | | - | 504 | 477 | - | - | 53 | 46 | - | - |
| Ordinal stroke or TIA (%) | 49 (5.0) | | 63 (6.2) | | 0.81  (0.55, 1.19) | | 0.28 | 41 (8.2) | 36 (7.6) | 1.16 (0.72, 1.87) | 0.53 | 3 (5.7) | 6 (13.0) | 0.33  (0.05, 2.35) | 0.27 |
| No stroke or TIA | 936 (95.0) | | 947 (93.8) | | - | | - | 461 (91.8) | 438 (92.4) | - | - | 50 (94.3) | 40 (87.0) | - | - |
| TIA | 17 (1.7) | | 25 (2.5) | | - | | - | 12 (2.4) | 18 (3.8) | - | - | 3 (37.5) | 5 (10.9) | - | - |
| mRS 0-1 | | 7 (0.7) | | 13 (1.3) | | - | - | 8 (1.6) | 5 (1.1) | - | - | 0 | 0 | - | - |
| mRS 2-3 | | 12 (1.2) | | 15 (1.5) | | - | - | 10 (2.0) | 7 (1.5) | - | - | 0 | 1 (2.2) | - | - |
| mRS 4-5 | | 6 (0.6) | | 5 (0.5) | | - | - | 5 (1.0) | 4 (0.8) | - | - | 0 | 0 | - | - |
| mRS 6 (death) | | 7 (0.7) | | 5 (0.5) | | - | - | 6 (1.2) | 2 (0.4) | - | - | 0 | 0 | - | - |
| Stroke (%) | | 32 (3.2) | | 38 (3.8) | | 0.85 (0.53, 1.38) | 0.52 | 29 (5.8) | 18 (3.8) | 1.65 (0.90, 3.05) | 0.11 | 0 | 1 (2.2) | NC |  |
| Death (%) | | 15 (1.5) | | 13 (1.3) | | 1.08 (0.50, 2.35) | 0.85 | 10 (2.0) | 15 (3.1) | 0.66 (0.28, 1.55) | 0.33 | 1 (1.9) | 0 | NC |  |
| Barthel Index | | 92.9 (19.6) | | 94.2 (17.4) | | -0.92 (-2.38, 0.55) | 0.22 | 91.0 (21.6) | 91.8 (19.9) | 0.21 (-2.20, 2.62) | 0.87 | 89.8 (22.0) | 95.5 (9.5) | -3.25 (-9.40, 2.91) | 0.30 |
| ZDS | | 45.4 (16.8) | | 45.9 (17.1) | | -0.31 (-1.87, 1.26) | 0.70 | 47.1 (17.1) | 48.2 (18.4) | -0.91 (-3.30, 1.48) | 0.46 | 50.1 (20.0) | 45.1 (16.9) | 3.21 (-4.07, 10.49) | 0.38 |
| EQ-5D-3L-HSUV | | 0.76 (0.31) | | 0.76 (0.29) | | 0.01 (-0.02, 0.03) | 0.68 | 0.74 (0.31) | 0.70 (0.32) | 0.03 (-0.01, 0.06) | 0.17 | 0.68 (0.33) | 0.80 (0.23) | -0.09 (-0.20, 0.01) | 0.08 |
| EQ-VAS | | 74.3 (21.0) | | 73.6 (21.6) | | 0.90 (-0.96, 2.75) | 0.34 | 70.4 (22.1) | 68.5 (24.0) | 1.66 (-1.26, 4.58) | 0.26 | 65.6 (23.2) | 74.0 (17.8) | -5.76 (-14.44, 2.93) | 0.19 |
| t-MMSE | | 18.5 (4.2) | | 18.7 (3.9) | | -0.22 (-0.60, 0.17) | 0.27 | 18.2 (4.2) | 17.7 (5.3) | 0.53 (-0.13, 1.19) | 0.11 | 17.3 (4.2) | 18.2 (4.2) | -0.65 (-2.67, 1.37) | 0.52 |
| TICS-M | | 21.4 (6.4) | | 21.7 (6.0) | | -0.24 (-0.83, 0.34) | 0.41 | 20.6 (6.0) | 19.8 (7.2) | 0.82 (-0.07, 1.72) | 0.07 | 19.3 (5.5) | 20.7 (6.1) | -0.73 (-3.41, 1.95) | 0.59 |
| Verbal fluency | | 17.3 (7.7) | | 17.6 (7.6) | | -0.30 (-1.01, 0.41) | 0.36 | 16.7 (7.3) | 15.9 (7.5) | 0.79 (-0.21, 1.80) | 0.12 | 15.3 (8.5) | 15.3 (6.9) | 0.92 (-2.74, 4.58) | 0.62 |
| Intracranial bleeding | | 7 (0.7) | | 4 (0.4) | | 1.84 (0.52, 6.50) | 0.34 | 9 (1.8) | 1 (0.2) | 12.09 (1.36, 107.18) | **0.025** | 0 | 0 | NC |  |
| Stroke or major/fatal bleeding | | 47 (4.8) | | 46 (4.6) | | 1.00 (0.61, 1.64) | 1.00 | 38 (7.6) | 22 (4.6) | 1.79 (0.91, 3.53) | 0.09 | 2 (3.8) | 1 (2.2) | NC |  |
| Death, stroke, myocardial infarction, or major bleeding | | 56 (5.7) | | 58 (5.7) | | 1.04 (0.66, 1.66) | 0.86 | 44 (8.8) | 39 (8.2) | 1.03 (0.60, 1.78) | 0.91 | 2 (3.8) | 1 (2.2) | NC |  |

Data are n (%), mean (standard deviation), mean difference (MD), odds ratio (OR) or common odds ratio (cOR) with 95% confidence intervals (CI). Comparison using binary or ordinal logistic regression or multiple linear regression. ACD: Aspirin, Clopidogrel, Dipyridamole; EQ-5D-3L-HSUV: European quality of life 5 dimensions 3 level health utility status value; EQ-VAS: European quality of life visual analogue scale; mRS: modified Rankin Scale; t-MMSE: telephone mini-mental state examination; TIA: transient ischaemic attack; TICS-M: telephone interview for cognition scale-modified; ZDS: Zung Depression Scale.

**The TARDIS Investigators**

A complete list of Investigators is provided in the Supplementary Appendix of the main publication (reference 6 in accompanying manuscript).

**Writing Committee**

Jason P Appleton, Lisa J Woodhouse, Maia Beridze, Hanne Christensen, Rob A Dineen, Timothy J England, Anna Ranta, Thompson Robinson, Nikola Sprigg; Philip M Bath, for the TARDIS Investigators

**Trial Steering Committee**

*Independent members*: Helen Rodgers (Newcastle, TSC Chair), Ahamad Hassan (Leeds), Christine Roffe (Stoke-on-Trent), Craig Smith (Salford), William D Toff (Leicester)

*Grant holders*: Philip Bath (Nottingham, Chief Investigator), Rob Dineen (Nottingham, Neuroradiology Lead), Lelia Duley (Nottingham), Stan Heptinstall (Nottingham, Platelet Expert), Marilyn James (Nottingham, Health Economic Lead), Hugh Markus (Cambridge), Stuart Pocock (London, Statistical Lead), Thompson Robinson (Leicester), Nikola Sprigg (Nottingham, Deputy Chief Investigator), Graham Venables (Sheffield)

*Patient-public representative (Nottingham)*: Oswald Newell (2008-14), Chibeka Kasonde (2014-16)

*Sponsor's representative*: Angela Shone (University of Nottingham)

**International Advisory Committee**

Denmark - Hanne Christensen (Copenhagen), Georgia - Maia Beridze (Tblisi), New Zealand - Anna Ranta (Wellington), UK – Philip Bath (Chair, Nottingham)

**Independent Data Monitoring Committee**

Ian Ford (Glasgow, UK; Chair), Didier Leys (Lille, France), Cathie Sudlow (Edinburgh, UK), Matthew Walters (Glasgow, UK)

**Events (outcome, SAE) Adjudicators**

Nikola Sprigg (Stroke Physician, Nottingham, UK), Marc Randall (Neurologist, Leeds, UK), Wayne Sunman (Stroke Physician, Nottingham, UK), Kailash Krishnan (Stroke Physician, Nottingham, UK)

**Neuroimaging Adjudicators**

Rob Dineen (Nottingham, UK), Alessandro Adami (Verona, Italy), Lesley Cala (Perth, Australia), Ana Casado (Edinburgh, UK), Rebecca Gallagher (Derby, UK), David Swienton (Leicester, UK), Satheesh Ramalingam (Birmingham, UK)

**Platelet substudy**

Stan Heptinstall, Sue Fox, Jane May (Nottingham, UK)
